# Notes on a Case of Lymphatic Leukæmia in a Child Aged Three Years

**Published:** 1914-06

**Authors:** Edward Cecil Williams

**Affiliations:** Physician, to the Royal Hospital for Sick Children and Women, Bristol


### NOTES ON A CASE OF LYMPHATIC LEUKÆMIA IN A CHILD AGED THREE YEARS.

BY

EDWARD CECIL WILLIAMS, B.A., M.B. Cantab.,

*Physician, to the Royal Hospital for Sick Children and Women, Bristol.*

THE lymphatic type is far and away the most common form of leukæmia met with in children. The following case is remarkable for the rapid progress of the acute symptoms (about seven days), and the extraordinary character of the terminal blood count, which showed the ratio of red cells to white cells to be 38.2 per cent. to 61.8 per cent.

J. B., male, aged 3 years, admitted September 17th, 1913, died September 18th, 1913.

*Family History.*—Mother died in child-birth. One other child living and healthy.

*Personal History.*—It is not definitely known whether he has had any infectious disease. Lumps have been noticed in the neck for the last six months. Child has got worse since the removal of tonsils and adenoids three months ago. Eyes became blackened a week ago.

*Condition on Admission.*—Perfectly conscious; hemorrhages into both eyelids; chest nil; enlarged glands in neck, axilla and groin.

*Abdomen.*—Liver enlarged two fingers' breadth below right costal margin; spleen enlarged to level of umbilicus; some free fluid in abdomen; some œdema of legs; no specimen of urine available for examination. T. 102.8, R. 48, P. 160.

September 18th, 1913.—6 a.m., T. 104° F., which quickly came down to 100° F., R. 32, P. 148, child sitting up in bed. 10 a.m., child comatose, hemorrhages over various parts of the body, some as large as a five shilling piece. 1 p.m., child died, comatose.

The following differential blood count was made in the Pathological Laboratory of the University of Bristol:—

| | | | |
|---|---|---|---|
| 3,000 cells counted. | *Abnormal mononuclears | 98.7 | per cent. |
| | Large mononuclears .. | 0.6 | ,, |
| | Polymorphs .. .. .. | 0.4 | ,, |
| | Eosinophiles .. .. .. | 0.2 | ,, |
| | Myelocytes .. .. .. | 0.6 | ,, |

Ratio of red cells to white cells, 38.2 per cent. to 61.8 per cent.

According to Gulland and Goodall, [1] in chronic cases the white cell count is much higher than in the acute cases, 500,000 white cells per cm. are not unknown in chronic

* The lymphocytes are quite unlike those seen in normal blood, the size is slightly larger than that of erythrocytes, most are spherical, but some show protuberances; the nucleus is large, vesicular, stains feebly, and is usually spherical, but sometimes bean-shaped; the cytoplasm in some is slightly granular. The whole cell has a tendency to smear. In appearance it is somewhat like the large mononuclear cells of normal blood. The percentage counts were given on counts made on the total number of cells in films counted from end to end in straight lines, commencing at one margin and proceeding to the opposite, the lines being taken at equal intervals to avoid errors in distribution as far as possible. These agree with the appearances shown by the blood in the vessels of the organs sectioned. No blood was available for direct counting by dilution in a hæmocytometer.—J. R. K.-M.

[1] Gulland and Goodall, *The Blood: a Guide to its Examination, etc.*, Edinburgh, 1912.

lymphæmia, but in acute cases the white cells seldom exceed 150,000 per cm. It is difficult, however, to average for the acute cases, as many are merely the acute terminations of chronic cases, and in them the count is naturally high. I have not, however, been able to find any record of a similar ratio of red cells to white cells to the above, even supposing this be only the terminal picture of a chronic case. The suggestion of Boycott,[1] that in this condition some of the lymphocyte-like cells are really erythroblasts in the stage before any hæmoglobin is developed in the cytoplasm, may to some extent explain the nearly two to one ratio of whites to reds.

REPORT ON THE TISSUES, REMOVED POST-MORTEM, BY PROFESSOR WALKER HALL AND DR. J. KAY-MOUAT.

The following tissues were most affected :—

*Nervous System.*—The entire brain was riddled with hemorrhages, from massive hemorrhages destroying large areas of brain to minute capillary hemorrhages.

*Kidneys.*—Tubular nephritis with fatty degeneration, desquamation and necrosis of a few cells in scattered areas. The tubules are widely separated by lymphocytes. Hemorrhages are frequent in the intertubular connective tissues, especially around the convoluted tubes. The glomeruli are engorged, but show no hemorrhages.

*Liver.*—Capillary channels widely distended with blood. Hemorrhages common in the portal systems and sometimes around the central vein. The cells adjoining some portal systems are filled with large globules of fat, a few have undergone fatty necrosis, most thus affected only show slight chromatolysis, but in some it is complete.

*Bone Marrow.*—Showed a proliferation of lymphocytes to such an extent as almost entirely to replace other types of cells.

*Lymphatic System and Glands.*—Lymphatics engorged with lymphocytes. *Lymphatic Glands.*—The nodules are indistinguishable, the whole of each gland is enlarged by proliferation of lymphocytes and endothelial cells, polykaryocytes are larger and more numerous than usual.

*Spleen.*—Malpighian corpuscles obliterated by general lymphocytic proliferation.

[1] Boycott, Article in *General Pathology*, edited by Pembrey and Ritchie, London, 1913.

*Thymus.*—Lymphoid nodules distinct, thus differing from lymphoid tissue elsewhere.

The following organs were comparatively healthy :—

*Lungs.*—Congestion, catarrh and hemorrhage, excess of lymphocytes.

*Heart.*—Myocardium shows no fragmentation or segmentation, cross striation well marked, no fatty degeneration or infiltration. The vessels are dilated, but there is a remarkable absence of cellular infiltration or lymphoid accumulations. There are no epicardial, myocardial or endocardial hemorrhages.

*Suprarenals,*—Congestion, no hemorrhages or lymphocytic deposits.

*Pancreas.*—Islands of Langerhans are well developed, the glandular cells are those of a normal active gland, no hemorrhages.

Lymphatic leukæmia may assume clinically three principal forms : (1) The anæmic, with tendency to late hemorrhages ; (2) the purpuric, with tendency to early hemorrhages ; (3) the scorbutic, in which lesions of the gums and stomatitis are leading features. The clue to the diagnosis lies in the microscopical examination of the blood, the enormous increase of lymphocytes in a film at once revealing the true nature of a case.

The outstanding features of this case are :—

1. The number and extent of the cerebral hemorrhages present in the white and grey matter of the entire brain.

2. Great proliferation of all lymphoid tissue, the thymus though large being of normal structure.

3. The healthy heart ; the absence of fatty degeneration being remarkable.

4. The great number of leucocytes in the blood, these cells actually out-numbering the erythrocytes.

The two forms of leukæmia, the myeloid and lymphatic, do not represent processes of essentially different kinds, but the obvious characteristics of each are sufficient to warrant their separation. There is, however, an increasing mass of evidence to support the theory that they represent a sarcoma of the leucoblastic and possibly of the erythroblastic portions of the bone marrow, rather than an infection. In support of this view may be mentioned: (1) It is impossible in the majority of cases of lymphatic leukæmia to distinguish the blood from that of chloroma; (2) lymphatic leukæmia is always fatal, this cannot be said of an infection; (3) the overgrowth of lymphocytes is useless, and useless overgrowth is practically identical, in its results, with tumour growth; (4) no constant toxin has been found in leukæmia.

I am indebted to Professor Walker Hall and Dr. J. Kay-Mouat for permission to use their pathological reports on the various organs made in the Pathological Department of the University.

